# MiR-92a and miR-486 are potential diagnostic biomarkers for mercury poisoning and jointly sustain NF-κB activity in mercury toxicity

**DOI:** 10.1038/s41598-017-13230-5

**Published:** 2017-11-22

**Authors:** Enmin Ding, Jun Guo, Ying Bai, Hengdong Zhang, Xin Liu, Wenyan Cai, Lixin Zhong, Baoli Zhu

**Affiliations:** 1 Institute of Occupational Disease Prevention, Jiangsu Provincial Center for Disease Prevention and Control, Nanjing, Jiangsu Province People’s Republic of China; 2 Yandu District Center for Disease Prevention and Control, YanCheng, Jiangsu Province People’s Republic of China; 3 School of Public Health, Nanjing Medical University, Nanjing, Jiangsu Province People’s Republic of China

## Abstract

Occupational and environmental exposure to mercury is a public health concern worldwide. Although the altered epigenetic regulatory features, such as microRNA, have been associated with mercury exposure, the underlying molecular mechanism is not well illuminated. This study aimed to confirm that hsa-miR-92a and hsa-miR-486 are novel diagnostic biomarkers of occupational mercury poisoning, and to explore the underlying mechanism of miR-92a and miR-486 in mercury toxicity. RT-qPCR assays and receiver operating characteristics curve analyses were conducted to confirm the diagnostic value of miR-92a and miR-486 as biomarkers of occupational mercury poisoning. Dual-luciferase assay was applied to confirm the target gene of miR-92a and miR-486 *in vitro*. Then, we established an *in-vitro* model where miR-92a and miR-486 were overexpressed or knocked down in HEK-293 and HUVEC cells. RT-qPCR and western blotting were used to analyze gene and protein expression levels. Cell apoptosis was determined by flow cytometry. Results show that miR-92a and miR-486 expression levels were up-regulated in workers exposed to occupational mercury. Upregulation of miR-92a and miR-486 may play a crucial role in mercury toxicity by jointly activating the NF-κB signaling pathway via targeting KLF4 and Cezanne, respectively.

## Introduction

Mercury (Hg) is one of the most widely used metals in industry, and is mainly used in mercury thermometer production and gold extraction. Mercury is a ubiquitous and persistent toxicant with pleiotropic effects. The inhalation of mercury vapor can produce harmful effects on the nervous system, the kidneys, and many other organic systems, including the gastrointestinal, respiratory, hepatic, immune, and integumentary system^1^. Recent studies provided an increasing amount of evidence that mercury exposure is associated with cardiovascular disease (CVD), including hypertension, arteriosclerosis and coronary heart disease^2–6^. Based on current studies, the assumed mechanism of mercury toxicity mainly involves its ability of binding to sulfydryl containing enzyme, increasing oxidative stress and reducing oxidative defense^7^. However, these mechanisms cannot fully explain the damage to numerous organic systems that has been associated with mercury.

Environmental and occupational toxicants such as mercury, lead and arsenic can alter epigenetic regulatory features such as DNA methylation, histone modification and microRNA (miRNA) expression pattern^8^. In animals, miRNAs are endogenous noncoding RNAs of approximately 23 nt, which play crucial gene-regulatory roles by binding to the 3′ UTR of protein-coding genes to mediate their posttranscriptional repression^9^. As circulating miRNAs are in a remarkably stable form that is protected from endogenous RNase, serum or plasma miRNAs have been widely used as a blood-based markers for diagnosis of human disease^10^. At present, the expression patterns of miRNA and their susceptibility to environmental factors is a focus of environmental epigenetics, with only a few publications illuminating the underling mechanism.

In our previous study, we identified plasma miRNAs hsa-miR-92a and hsa-miR-486 as potential markers to diagnose occupational mercury poisoning in workers^11^. In the present study, we further validate this result in a larger sample group, and investigate the change of plasma levels of miR-92a and miR-486 over time. Finally, we explore the underlying mechanism of miR-92a and miR-486 in mercury toxicity.

## Materials and Methods

### Study population

This research has been approved by the Jiangsu Provincial Center for Disease Prevention and Control ethics committee and all the experiments were carried out in accordance with relevant guidelines and regulations. Written informed consent was acquired from all of the subjects who participated in our research. We recruited all subjects from one mercury thermometer factory located in Eastern China. Urine and whole blood of subjects were collected at the same day when they had their occupational health examination in 2013. Inclusion criteria were as follow: *a)* person who has worked in the mercury thermometer plant for at least 1 year, and *b)* person who was exposed to mercury at work. Exclusion criteria were as follow: *a)* person with chronic disease history, including primary renal and nervous disease, *b)* person who has taken any medicines in the preceding 3 months, and *c)* person who did not provide urine and/or blood samples. Urine Hg was determined by cold vapor atomic absorption spectrometry (CVAAS, method II), using reduction with stannous chloride, according to the national standard of China^12^. Based on the research objectives, the subjects were divided into two groups: Hg-poisoning and Hg-absorbing. We defined Hg-poisoning and Hg-absorbing according to the Diagnostic Criteria of Occupational Mercury Poisoning of China (GBZ 89-2007). Workers who had increased urine mercury (over 35 μg/g Cr) and any three of the following clinical symptoms: *a)* neurasthenia, *b)* stomatitis or gingivitis, *c)* tremor, and *d)* proximal renal tubular dysfunction, were classified as Hg-poisoning group. Workers with increased urine mercury, but no clinical symptoms, were assorted as Hg-absorbing group. People working in the offices with urine mercury less than 4 μg/g Cr (the normal reference value of urine Hg of Chinese people) were selected as control group. All of these subjects were matched by age, sex, year of Hg exposure, smoking and drinking status. All the participants are Han Chinese people. Then, we followed these workers in 2014 and 2015. All blood and urine samples were stored at −80 °C.

### Cell culture

HEK-293 and Human umbilical vein endothelial (HUVEC) cells (all purchased from ATCC) were cultured in DMEM (Invitrogen, USA) supplemented with 10% fetal bovine serum (FBS). Cell survival rates were determined by MTT assay. Mercuric chloride was purchased from Sigma-Aldrich (USA).

### Knockdown and overexpression of miR-92a and miR-486

We used inhibitors of miR-92a and miR-486 (anti-92a and anti-486, 100 nmol/L) to knock down miR-92a and miR-486. Overexpression of miR-92a and miR-486 was achieved by transfecting cells with miR-92a and miR-486 mimics (miR-92a and miR-486, 50 nmol/L). MiR-92a and miR-486 mimics, mimic controls, inhibitors, and inhibitor controls were purchased from Ribo (Guangzhou, China). Transfections were performed using lipofectamine 2000 (Invitrogen, USA).

### Knockdown of COX-2 in HEK-293 and HUVEC cells

Two different COX-2 siRNA fragments and siRNA NC were designed and synthesized by Ribo (Guangzhou, China); the sequences were as follows: siRNA1, target sequence: GCTGGGAAGCCTTCTCTAA, sense 5′-GCUGGGAAGCCUUCUCUAAdTdT-3′, antisense 3′-dTdTCGACCCUUCGGAAGAGAUU-5′; siRNA2, target sequence: GGACTTATGGGTAATGTTA, sense 5′-GGACUUAUGGGUAAUGUUAdTdT-3′, antisense 3′-dTdTCCUGAAUACCCAUUACAAU-5′. HEK-293 and HUVEC cells were transfected separately with the two siRNAs and siRNA NC (50 nmol/L) by Lipofectamine 2000 (Invitrogen, USA) to knockdown the COX-2.

### MicroRNA extraction

Total plasma RNA was extracted from 200 μl plasma sample of each person by using the miRNeasy Serum/Plasma Kit (Qiagen, Germany). Synthetic Caenorhabditis elegans miRNA 238 (*cel-miR-238*) (Takara, Dalian, China) was added to each plasma sample prior to the isolation procedure as control. Plasma miRNA levels were quantified with the 2^−ΔΔCt^ (ΔΔCt = (Ct _miRNA 1_ − Ct _*cel-miR-238*_) − (Ct _miRNA2_  − Ct _*cel-misR-238*_)) relative quantification method using *cel-miR-238* as control. Total RNA of cultured cell was extracted using Trizol (Invitrogen, Carlsbad, CA, USA). Cell miRNA levels were quantified with the 2^−ΔΔCt^ (ΔΔCt = (Ct _miRNA 1_ − Ct _*U6*_)  − (Ct _miRNA2_  − Ct _*U6*_)) relative quantification method using human U6 as internal control.

### RT-qPCR analysis

cDNA was synthesized using TaqMan miRNA-specific stem-loop primers and the TaqMan® miRNA Reverse Transcription Kit (Applied Biosystems, USA), and amplified by PCR. SYBR-green reverse transcription PCR (RT-qPCR) was performed to detect Krüppel-like factor 4 (KLF4), Cezanne, nuclear factor-κB (NF-κB), cyclooxygenase-2 (COX-2) and GAPDH mRNA levels using the SYBR Green PCR Kit (Applied Biosystems, USA). Total RNA was prepared from the cells 24 h after Hg treatment and transfection, followed by RT-qPCR analysis. The sequences and amplification efficiency of each primer can be found in the Supplementary Tables S1–S3.

### Western blot analysis

Antibodies against KLF4, Cezanne, NF-κB, COX-2, GAPDH, and secondary antibodies were obtained from Abcam (USA). Primary antibodies and secondary antibodies used for Western blot analyses were diluted 1:1,000 in 5% defatted milk as a working concentration. Western blot analysis was conducted according to the guidelines and instructions of Abcam. GAPDH was used as loading control. Total protein was prepared from the cells 48 h after Hg treatment and transfection, followed by Western blot analysis. 20 μg protein of each sample was loaded onto the SDS-polyacrylamide gels (BIO-RAD). Polyvinylidene-fluoride membrane (0.45 μm) were purchased from and Millipore.

### KLF4 and Cezanne 3′ Untranslated Region Luciferase Assay

For validation of KLF4 and Cezanne as targets of miR-92a and miR-486, respectively, wild-type and mutant-type 3′ untranslated region of human KLF4 and Cezanne were cloned into a *Renilla* luciferase control reporter vector (pRL-TK; Promega, USA). HEK-293 and HUVEC cells were co-transfected with pRL-TK vectors, pGL3 vector control, miR-92a, miR-486 mimics or mimic-negative controls, and miR-92a, miR-486 inhibitors or inhibitor negative controls, using lipofectamine® 2000 (Invitrogen, USA). Firefly luciferase activity was detected using dual-luciferase assays (Promega), which were normalized to a *Renilla* luciferase expression control.

### Cell apoptosis assay by flow cytometry (FCM)

For flow cytometry, HEK-293 and HUVEC cells were harvested 48 h after Hg treatment and transfection. Cell apoptosis assays were conducted according to the Annexin V-FITC staining protocol by using the ANNEXIN V-FITC APOPTOSIS DETECTION KIT I and BD Accuri C6 flow cytometry (BD Biosciences, USA).

### Statistical analysis

All statistical analyses were carried out using SPSS 21.0 software. *P*-values were determined by ANOV for continuous variables and by chi-square test for categorical variables. *LSD* were used for multiple comparison in ANOV. Urine mercury underwent a log10-transformation for statistical analysis. The relative expression level of each miRNA was individually calculated by 2^−ΔΔCt^ (ΔΔCt = (Ct _miRNA 1_ − Ct _*control*_)  − (Ct _miRNA2_  − Ct _*control*_)). The relative quantification value then underwent a log2-transformation to compare the expression levels of the candidate miRNAs between groups. Receiver operating characteristics (ROC) curve analyses and the area under the curve (AUC) analyses were used to evaluate the diagnostic value of miR-92a and miR-486. One-way ANOVA analysis was used to analyze the expression levels of miRNAs, gene and protein levels. In all cases, *p* < 0.05 was considered statistically significant.

## Results

### General characteristics of study subjects

As shown in Table 1, the distribution of age, sex, smoking, drinking and working years were matched between A) mercury poisoning group, B) mercury absorbing group and C) control group (all *p* > 0.05), whereas the urine mercury levels were all significantly different between three groups (*p* < 0.001).

**Table 1:** General characteristics of study subjects

Variable	Mercury poisoning group (n = 30)	Mercury absorbing group (n = 30)	Control group (n = 30)	*P*
Age (years)	37.57 ± 6.82	36.27 ± 7.42	37.94 ± 6.69	0.792
Sex (% female)	86.7	86.7	86.7	1.000
Smoking (yes/no)	0/30	0/30	0/30	1.000
Drinking (yes/no)	0/30	0/30	0/30	1.000
No. of working years	19.67 ± 6.99	18.77 ± 7.15	17.00 ± 7.69	0.645
Urine mercury (μg/g Cr)	685.45 (233.90~1363.25)	53.15 (45.65~67.43)	0.80 (0.67~0.93)	<0.001

Ages and working years were shown are mean ± SD; urine mercury was shown as median (P_25_~P_75_).

### MiR-92a-3p and miR-486-5p are up-regulated in mercury-exposed workers

**Figure 1:**
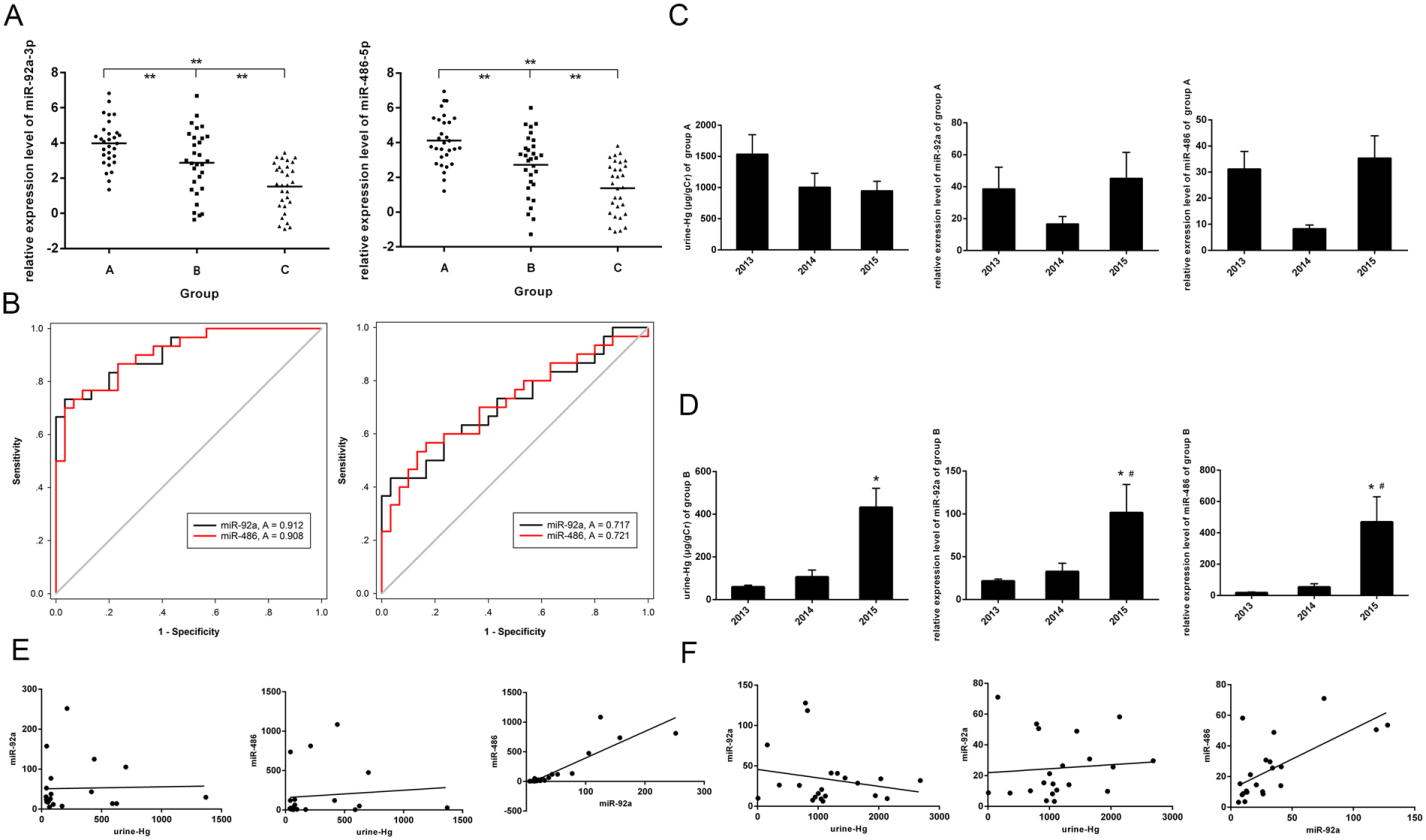
MiR-92a-3p and miR-486-5p are up-regulated in mercury exposed workers. (**A**) miR-92a and miR-486 expression levels were determined by Taqman RT-qPCR in mercury poisoning (group A), mercury absorbing (group B) and control group (group C). (**B**) ROC and AUC were used to evaluate the correlation of miR-92a and miR-486 levels with mercury poisoning and mercury absorption. Urine Hg, miR-92a and miR-486 levels of mercury poisoning (**C**) and mercury absorbing (**D**) workers were detected in 2013, 2014 and 2015. Data are presented as mean ± SE and analyzed by ANOV. Scatter diagrams of the correlationship between urine Hg, miR-92a and miR-486 of mercury poisoning (**E**) and mercury absorbing (**F**) group were drew. **p < 0.001, compared with control group. *p < 0.05, compared with 2013. ^#^ *p* < 0.05, compared with 2014.

As shown in Fig. 1A, expression of miR-92a and miR-486 was significantly up-regulated in workers with mercury poisoning and mercury absorbing workers compared with the control group (*P* < 0.001). Next, we used receiver operating characteristics (ROC) curve analysis, and calculated the area under the curve (AUC), to evaluate the correlation of miR-92a and miR-486 levels with mercury poisoning and mercury absorption (Fig. 1B). The AUC of miR-92a and miR-486 for workers with mercury poisoning were 0.912 and 0.908, respectively. The AUC of miR-92a and miR-486 for mercury absorbing workers were 0.717 and 0.721, respectively. The diagnostic value of miR-92a is higher than miR-486 for both mercury poisoning and mercury absorption. Subsequently, we sampled urine and blood from the same workers for the next two years. We determined urine Hg, and the expression levels of miR-92a and miR-486 for the group with mercury poisoning and the mercury-absorbing group, as shown in Fig. 1C and D. Scatter diagrams of urine Hg, miR-92a and miR-486 were drew and shown in Fig. 1E and Fig. 1F. Spearman correlation was used to calculate the R^2^ and p value of coefficient. The R^2^ of correlation between urine Hg and miR-92a was 0.001 and 0.041 for mercury absorbing and poisoning group (*P* = 0.91 and 0.38). The R^2^ of correlation between urine Hg and miR-486 was 0.010 and 0.008 for mercury absorbing and poisoning group (*P* = 0.67 and 0.71). The R^2^ of correlation between urine miR-92a and miR-486 was 0.767 and 0.446 for mercury absorbing and poisoning group (*P* < 0.001). The relative expression levels of between miR-92a, miR-486 and urine Hg were not statistically correlated.

### MiR-92a and miR-486 are up-regulated in Hg-treated (HgT) cells

**Figure 2:**
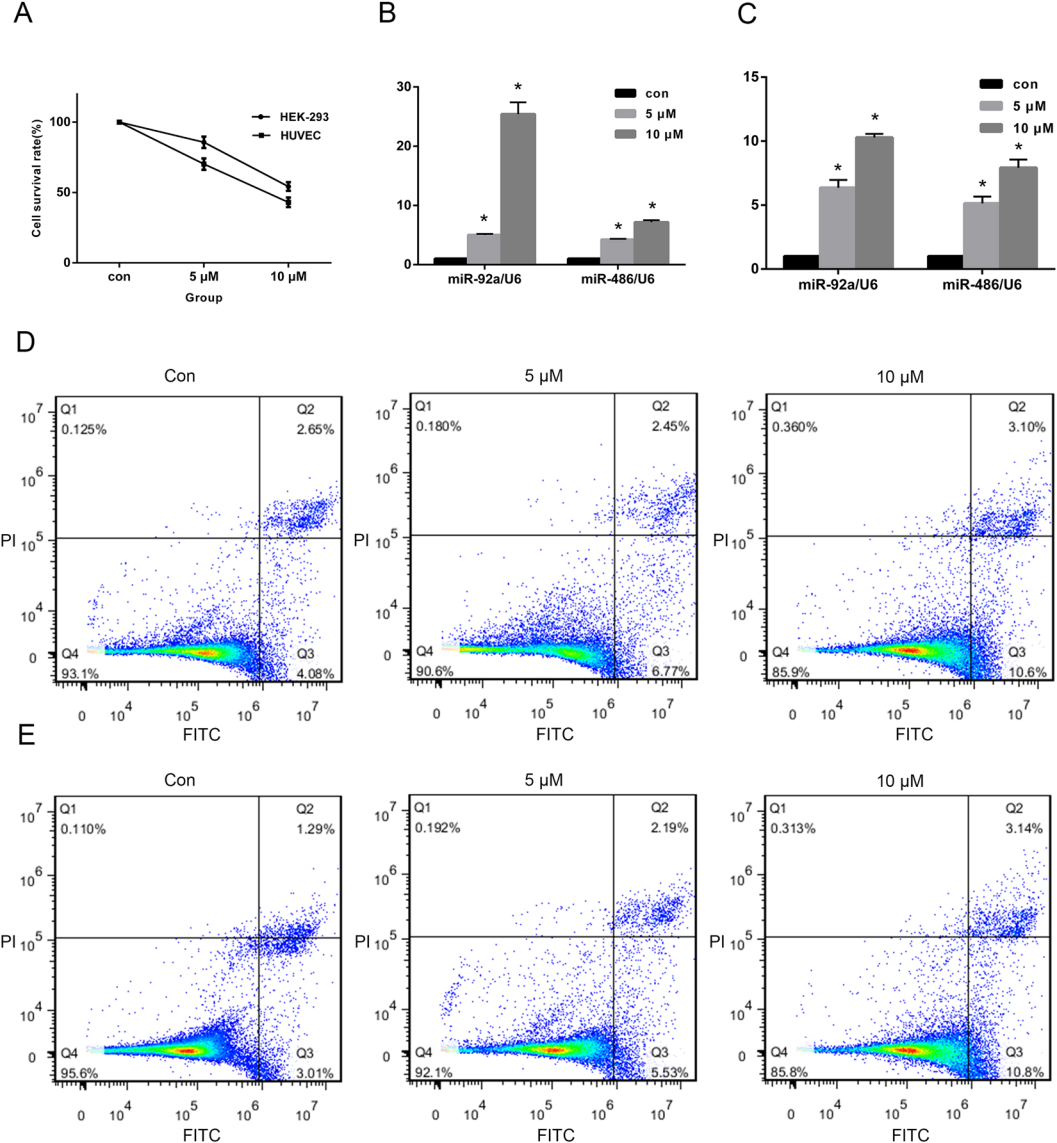
MiR-92a and miR-486 are up-regulated in Hg-treated (Hg-T) cells. (**A**) The survival rates of HEK-293 and HUVEC cells with mercuric chloride at 5 μM and 10 μM were determined by MTT assays. (**B**) HEK-293 and HUVEC cells were treated with mercuric chloride at different concentrations for 24 hr. miR-92a and miR-486 expression levels were determined by RT-qPCR. Data represent mean ± SE from three replicates from each treatment and were analyzed by one-way ANOVA. Apoptosis rates of Hg-T HEK-293 (**C**) and HUVEC (**D**) cells were determined by flow cytometry. *p < 0.05, compared with control.

In order to investigate whether mercury could induce the change of miR-92a and miR-486 expression levels *in vitro*, HEK-293 and HUVEC cells were treated with mercuric chloride at different concentrations for 24 h. Exposure doses were determined by MTT assays. As shown in Fig. 2A, the survival rates of HEK-293 and HUVEC cells exposed to 10 μM mercuric chloride were 54.28% and 43.03%, respectively. Subsequent RT-qPCR analysis revealed that miR-92a and miR-486 were highly up-regulated in HEK-293 and HUVEC cells (Fig. 2B). Flow cytometry analysis indicated that apoptosis rates of HEK-293 and HUVEC cells were significantly increased after treatment with 5 μM or 10 μM mercuric chloride, compared to the control group (Fig. 2C and D, respectively).

### MiR-92a-3p directly targets KLF4 and sustains NF-κB activity

**Figure 3:**
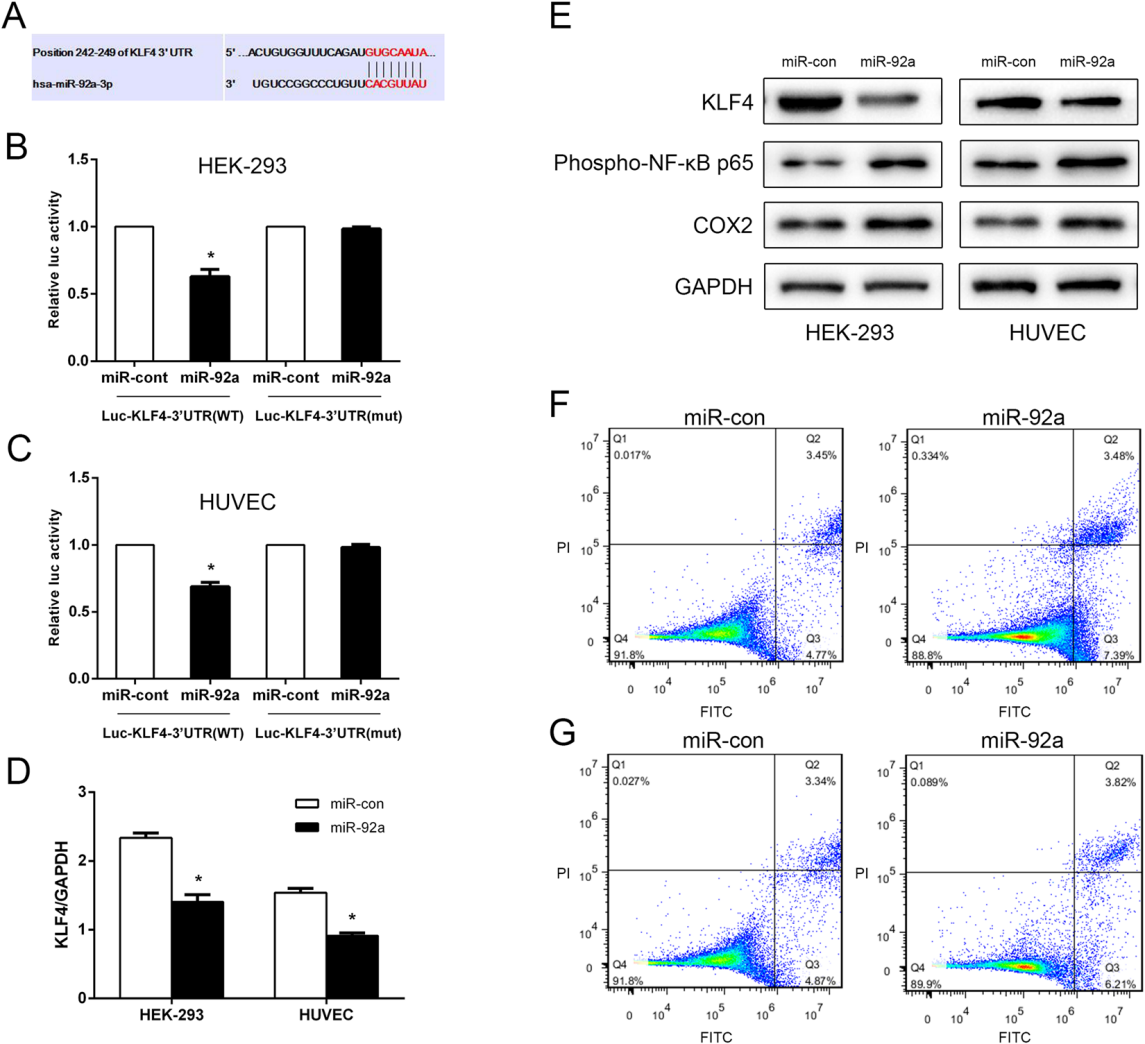
MiR-92a-3p directly targets KLF4 and sustains NF-κB activity. (**A**) Predicted miR-92a target sequence in the 3′UTR of KLF4 (KLF4-3′UTR). Luciferase activity of wild-type KLF4-3′-UTR (WT) or mutant KLF4-3′-UTR (mut) together with miR-con or miR-92a transduced HEK-293 (**B**) and HUVEC (**C**) cells. Each bar represents the mean ± SE of three independent experiments. (**D**) Real-time PCR analysis of KLF4 expression in miR-92a or miR-con transduced cells. Each bar represents the mean ± SE of KLF4/GAPDH of three independent experiments. (**D**) Western blot analysis of KLF4, phospho-NF-κB p65 and COX-2 protein levels in miR-con or miR-92a transduced cells. GAPDH served as the loading control. Apoptosis rates of miR-con or miR-92a transduced HEK-293 (**F**) and HUVEC (**G**) cells were determined by flow cytometry. *p < 0.05, compared with control.

MicroRNA target prediction (TargetScan, miRANDA) showed that KLF4 is the predicted target of miR-92a-3p (Fig. 3A). To explore whether miR-92a definitely targets KLF4, HEK-293 and HUVEC cells were co-transfected with a *Renilla* luciferase reporter vector containing the wild-type KLF4-3′-UTR (WT) or mutant KLF4-3′-UTR, together with 10 nmol/L miR-92a or a miRNA control. A dual-luciferase reporter gene assay showed that luciferase activity in KLF4 3′-UTR (WT) transfected cells decreased in presence of miR-92a, whereas an inhibitory effect of miR-92a on the luciferase expression in cells containing mutant KLF4-3′-UTR (mut) was not detected (Fig. 3B and C). Thus, our results demonstrate that KLF4 is a definite target of miR-92a *in vitro*. Next, miR-92a was overexpressed in HEK-293 and HUVEC cells to investigate the effects of miR-92a on KLF4 expression, and on its downstream signal molecules (NF-κB and COX-2). RT-qPCR confirmed a decreased level of KLF4 mRNA in miR-92a-transfected cells compared with the miRNA control-transfected HUVEC and HEK-293 cells (Fig. 3D). Western blot analysis revealed that the protein levels of KLF4 decreased, whereas phospho-NF-κB p65 and COX-2 increased in miR-92a-transfected cells compared with the control cells (Fig. 3E). Results of a cell apoptosis assay by FCM showed that apoptotic rates increased in miR-92a-transfected cells compared with the controls (Fig. 3F for HEK-293 and Fig. 3G for HUVEC).

### MiR-486-5p directly targets Cezanne and activates NF-κB signaling

**Figure 4:**
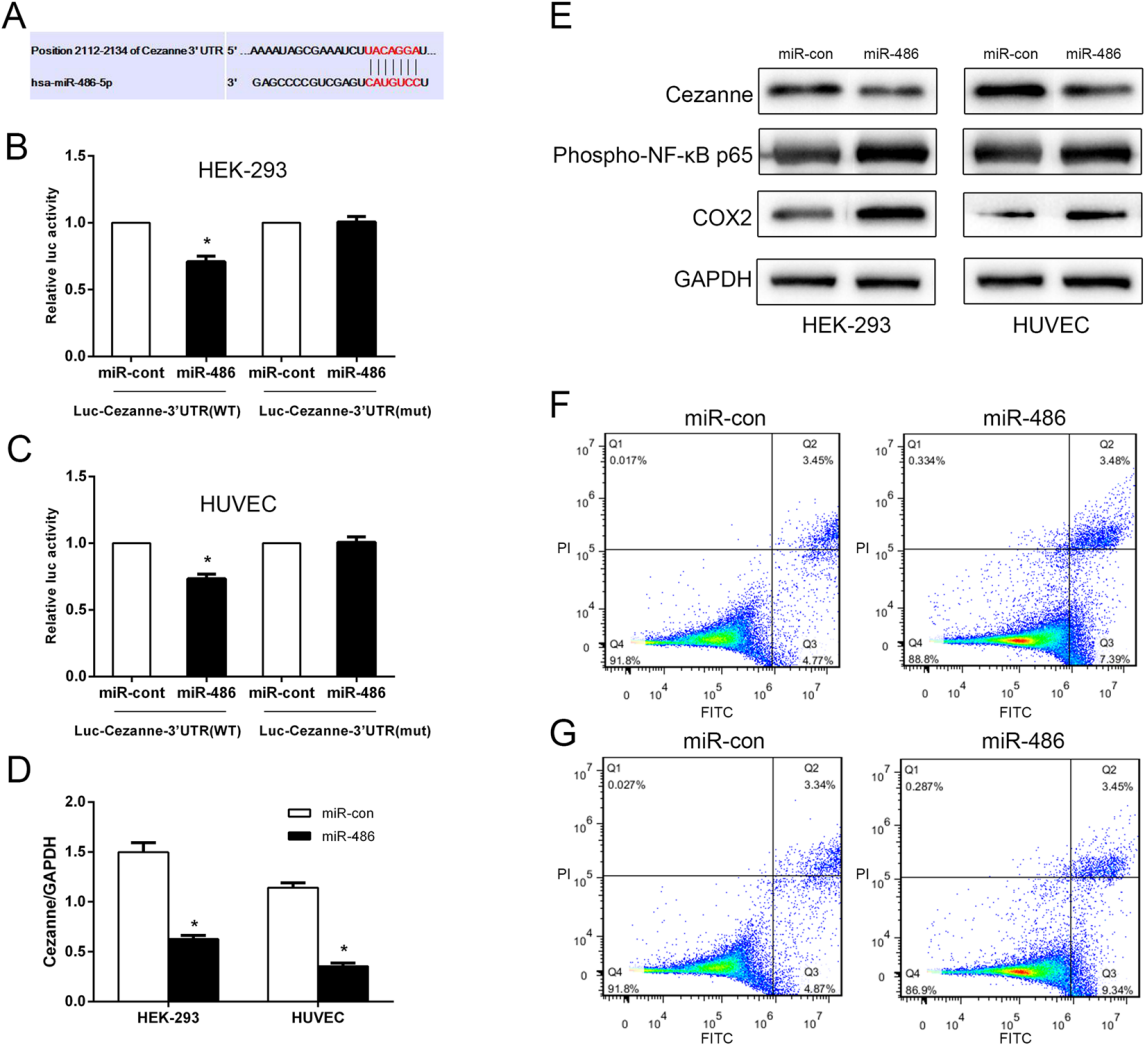
MiR-486-5p directly targets Cezanne and sustains NF-κB activity. (**A**) Predicted miR-486 target sequence in the 3′UTR of Cezanne (Cezanne -3′UTR). Luciferase activity of wild-type Cezanne -3′-UTR (WT) or mutant Cezanne -3′-UTR (mut) together with miR-con or miR-486 transduced HEK-293 (**B**) and HUVEC (**C**) cells. Each bar represents the mean ± SE of three independent experiments. (**D)** Real-time PCR analysis of Cezanne expression in miR-486 or miR-con transduced cells. Each bar represents the mean ± SE of Cezanne/GAPDH of three independent experiments. (**E**) Western blot analysis of Cezanne, phospho-NF-κB p65 and COX-2 protein levels in miR-con or miR-486 transduced cells. GAPDH served as the loading control. Apoptosis rates of miR-con or miR-486 transduced HEK-293 (**F**) and HUVEC (**G**) cells were determined by flow cytometry. *p < 0.05, compared with control.

Based on bioinformatic microRNA target analysis (TargetScan, miRANDA), *Cezanne* is the predicted target for miR-486-5p (Fig. 4A). HEK-293 and HUVEC cells were co-transfected with the wild-type Cezanne-3′-UTR (WT) or mutant Cezanne-3′-UTR (mut) together with 10 nmol/L miR-486 or miRNA control to prove that miR-486 directly targets *Cezanne*. The dual-luciferase reporter gene assay showed luciferase activity in cells transfected with Cezanne 3′-UTR (WT) decreased in presence of miR-486, whereas an inhibitory effect of miR-486 on the luciferase expression in cells with mutant Cezanne -3′-UTR (mut) was not detected (Fig. 4B and C). Thus, our results demonstrate that Cezanne is a definite target for miR-486 *in vitro*. Next, miR-486 was overexpressed in Hg-T (10 μM) HEK-293 and HUVEC cells to investigate the effects of miR-486 on Cezanne expression and its downstream signal molecules (NF-κB and COX-2). RT-qPCR was performed and confirmed the decreased level of Cezanne mRNA in miR-486-transfected cells compared with the miRNA control-transfected HUVEC and HEK-293 cells (Fig. 4D). Western blot analysis revealed that the protein level of Cezanne decreased, whereas phospho-NF-κB p65 and COX-2 increased in miR-486-transfected cells compared with the control cells (Fig. 4E). Results of the cell apoptosis assay by FCM showed that early apoptotic rates increased in miR-486-transfected cells compared with the controls (Fig. 4F for HEK-293 and Fig. 4G for HUVEC).

### MiR-92a and miR-486 induce apoptosis by jointly activating NF-κB and COX-2

**Figure 5:**
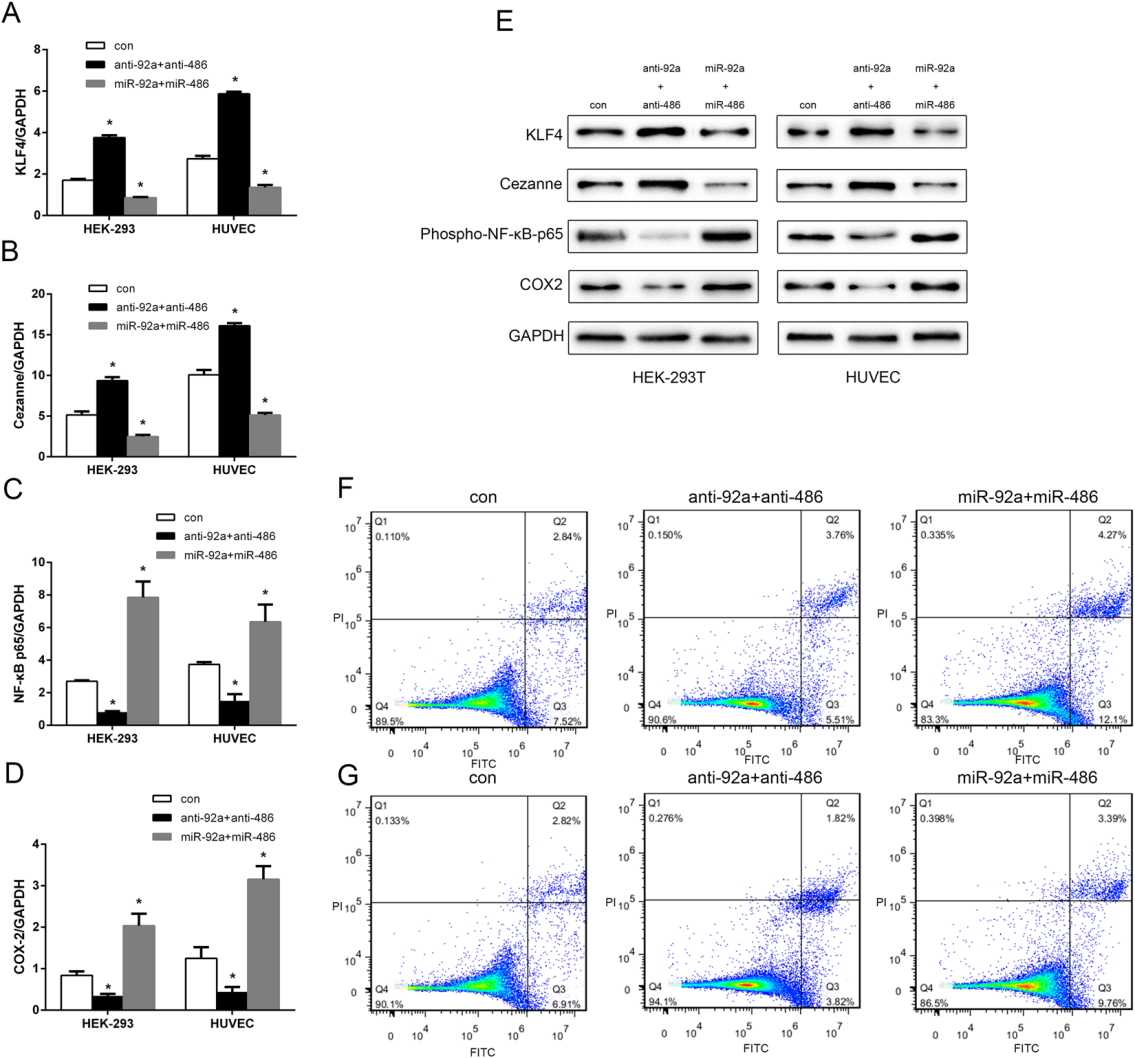
MiR-92a and miR-486 induce apoptosis by jointly activating NF-κB and COX-2. RT-qPCR analysis of relative KLF4 (**A**), Cezanne (**B**), NF-κB p65 (**C**) and COX-2 (**D**) expression level in miR-92a and miR-486 or anti-92a and anti-486 transfected cells compared with Hg-T cell. Each bar represents the mean ± SE of mRNA/GAPDH of three independent experiments. (**E**) Western blot analysis of KLF4, Cezanne, phospho-NF-κB p65 and COX-2 protein levels in miR-92a and miR-486 or anti-92a and anti-486 transduced cells. GAPDH served as the loading control. Apoptosis analysis of miR-92a and miR-486 or anti-92a and anti-486 transduced HEK-293 (**F**) and HUVEC (**G**) cells. *p < 0.05, compared with control.

In the previous experiments, KLF4 and Cezanne were shown to be the definite targets of miR-92a and miR-486, respectively. Interestingly, numerous studies have reported that KLF4 and Cezanne are both negative regulators of NF-κB, and that inhibition of miR-92a and miR-486 could reduce the expression of the active form of NF-κB, phospho-p65^13^, an important promoter of inflammation^14^. RT-qPCR and Western blot analysis were conducted to prove that miR-92a and miR-486 jointly activate NF-κB, and that inhibition of miR-92a and miR-486 could reduce the expression of NF-κB in Hg-T (10 μM) cells. Hg-T HEK-293 and HUVEC cells were transfected with 10 nmol/L miR-92a and miR-486 or 20 nmol/L anti-92a and anti-486. We observed that mRNA and protein levels of KLF4 and Cezanne in miR-92a and miR-486 transfected cells were decreased compared to levels in Hg-T cells, but increased in anti-92a and anti-486 transfected cells (Fig. 5A and B). In addition, phospho-NF-κB p65 and COX-2 mRNA and protein levels were markedly increased in miR-92a and miR-486 transfected cells compared to levels in Hg-T cells; however, they were decreased in anti-92a and anti-486 transfected cells (Fig. 5C–E). Cell apoptosis analysis showed that apoptotic rates increased in miR-92a and miR-486 transfected cells compared with Hg-T cells. In contrast, anti-92a and anti-486 could reduce the cell apoptotic rates compared with miR-92a and miR-486 transfected cells (Fig. 5F for HEK-293 and Fig. 5G for HUVEC).

### Knockdown of COX-2 in Hg-T cells could attenuate Hg-induced apoptosis

**Figure 6:**
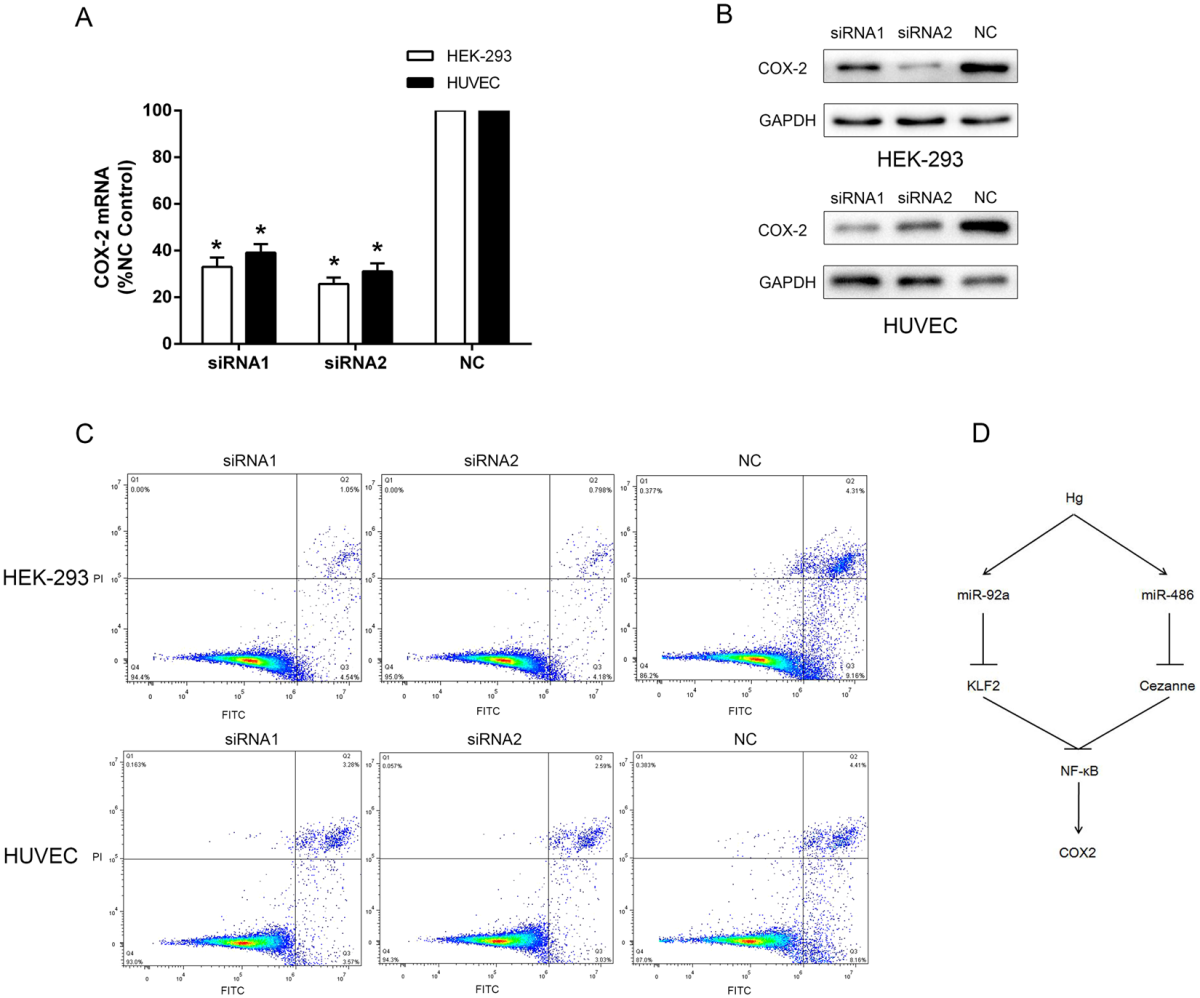
Knockdown of COX-2 in Hg-T cells could attenuate Hg-induced apoptosis. Analysis of COX-2 mRNA (**A**) and protein (**B**) levels in siRNA1and siRNA2 transfected Hg-treated HEK-293 and HUVEC cells compared to control cells. (**C**) Apoptosis rates of siRNA transduced Hg-T HEK-293 and HUVEC cells were determined by flow cytometry. *p < 0.05, compared with control.

As shown in Fig. 6A and B, COX-2 mRNA and protein levels were markedly reduced in siRNA1 and siRNA2-COX-2 transfected HEK-293 and HUVEC cells compared to levels in Hg-T cells. In Fig. 6C, siRNA1 and siRNA2 of COX-2 could reduce the Hg-induced apoptotic rates of HEK-293 and HUVEC cells compared with the controls.

## Discussion

The associations between the aberration of miRNAs profiles and heavy metals exposure have been confirmed by a number of studies^15,16^. Our population-based study confirmed that miR-92a and miR-486 expression levels were upregulated in workers exposed to occupational mercury. Subsequent experimental research revealed that exposure to mercury induced an increase in miR-92a and miR-486 expression, which led to increased NF-κB and COX-2 activation and apoptosis of cells.

NF-κB signaling plays a critical role in mediating the expression of numerous proinflammatory gene products, including COX-2 and inducible nitric oxide synthase (iNOS), which are involved in inflammatory responses^17^. Consistent with our findings, Park *et al*.^17^ reported recently that mercury could induce NF-κB activation, resulting in increased expression of COX-2. However, previous research did not thoroughly illuminate the relationship between mercury exposure and NF-κB activation. Here, we demonstrate that miR-92a and miR-486 may be the bridge between mercury exposure and NF-κB activation.

In this study, miRNA target prediction and experimental results identified KLF4 and Cezanne as direct targets for miR-92a and miR-486, respectively, which was consistent with findings reported previously^18,19^. Interestingly, KLF4 and Cezanne have both been reported to be important negative regulators of NF-κB signaling, and they repress phospho-NF-κB-p65 (the active form of NF-κB) through different mechanisms. KLF4, one of the zinc-finger transcription factors that mediate inflammation, can interact with phospho-NF-κB-p65 and promote COX-2 activity^20^. However, Cezanne, a newly identified member of the A20 family of deubiquitinases, suppresses NF-κB activity by deconjugating K63-polyubiquitin chains from RIP1 and TRAF6^19^. Consistently, our research demonstrated that overexpression of miR-92a and miR-486 can jointly increase NF-κB activity and COX-2 expression by targeting KLF4 and Cezanne, respectively, whereas inhibition of miR-92a and miR-486 showed the opposite effects.

MiR-92a, possibly originated from endothelial cells, has been proven to play a predominant role in re-endothelialization, angiogenesis, and inflammation^21,22^. It is also an important regulator of atherosclerosis development through endothelial activation^23^. Thus, previous studies together with our results suggest that miR-92a may a play crucial role in mercury-driven hypertension, atherosclerosis and other cardiovascular diseases. A recent study showed that miR-486 is a critical mediator involved in NF-κB activation, which contributes to glioma progression^19^. COX-2 was found to be markedly upregulated in brain disease and could induce apoptosis through its downstream product prostaglandin E_2_ (PGE_2_)^24,25^. All of the previous evidence combined with our results was helpful to explain the neurotoxicity resulting from mercury exposure. However, the mechanisms by which miR-92a and miR-486 are upregulated in mercury exposed workers are still unknown and currently under investigation in our laboratory.

Future studies will include follow up on the workers investigated in this study to observe the effects of occupational mercury exposure on the cardiovascular system, and aim to identify the underlying mechanism. In addition, there was no statistical correlation between the relative expression levels of miR-92a, miR-486 and urine Hg over time. The expression pattern of plasma miR-92a and miR-486 of mercury exposed workers over time merits future research. In our study, only one target was selected for validation for each miRNA, thus validation of more targets of miR-92a and miR-486 and exploration of the role of these targets is one of the important directions of future research. Last, males only make up just 13.3% of all the subjects, conclusions of this population study may be only appropriate for female workers exposed to mercury. Future study including a larger proportion of male is needed to prove our results.

## Conclusions

The present study provides a novel mechanism of mercury toxicity and may be the first study in which the role of miRNA in mercury toxicity is well illustrated. As shown in Fig. 6D, exposure to mercury could induce the upregulation of miR-92a and miR-486 which results in reductions of KLF4 and Cezanne. In addition, reductions of KLF4 and Cezanne jointly induce NF-κB activation and COX-2 expression, which eventually cause apoptosis. These combined regulatory effects of miR-92a and miR-486 in NF-κB activation may have crucial roles in mercury toxicity, and may have clinical implication in targeted therapy for mercury-induced poisoning in the future. However, these conclusions need to be confirmed in target tissues and organs of mercury poisoning patients in future studies. In conclusion, our research shows the aberrant expressions of miR-92a and miR-486 in mercury poisoning and both miRNAs may play crucial roles in mercury toxicity by activating NF-κB signaling.

## Electronic supplementary material

Supplementary information
