# Supplementary material for: MiR-92a and miR-486 are potential diagnostic biomarkers for mercury poisoning and jointly sustain NF-κB activity in mercury toxicity
Source: Sci Rep. 2017 Nov 22;7:15980. doi: 10.1038/s41598-017-13230-5 (PMC5700070; doi:10.1038/s41598-017-13230-5)
Supplement: Supplementary file 1 — Supplementary information [file 41598_2017_13230_MOESM1_ESM.pdf]

MiR-92a and miR-486 are potential diagnostic biomarkers for mercury poisoning and jointly sustain NF-κB activity in mercury toxicity

*Enmin Ding,[1] Jun Guo,[2] Ying Bai,[1] Hengdong Zhang,[1] Xin Liu,[1] Wenyan Cai,[3] Lixin Zhong,[1] and Baoli Zhu[1]**

Supplementary Table S1. Primers used for quantitative real-time PCR (qRT-PCR)

	F-primer	R-primer	OD	Final Conc.(μM)
GAPDH	5'-CATGAGAAGTATGACAACAGCCT-3'	5'-AGTCCTTCCACGATACCAAAGT-3'	5.0	20
NF-κB	5'-AGTTGAGGGGACTTTCCCAGGC-3'	5'-GCCTGGGAAAGTCCCCTCAACT-3'	5.0	20
COX2	5'-AATGAGTACCGAAATTC-3'	5'-CATCTAGTCCGGACCGGGAAG-3'	5.0	20
KLF4	5'-GCGGGAAGGGAGAAGACACT-3'	5'-GGGGAAGACGAGGATGAAGC-3'	5.0	20
Cezanne	5'-ACTGCCGAGGAATGAAGAGA-3'	5'-GCCAGCTGGATGTATGGTAGA-3'	5.0	20

Supplementary Table S2. Statistical analysis for amplification efficiency of mRNA primers

Primer	Slope	95%CI	Constant	Amplification efficiency
GAPDH	-1.128	-1.275~-0.981	15.710	0.85
NF-κB	-1.105	-1.253~-0.958	20.551	0.87
COX2	-1.060	-1.222~-0.898	25.210	0.92
KLF4	-1.044	-1.173~-0.919	20.914	0.94
Cezanne	-1.088	-1.144~-1.032	20.297	0.89

There is no statistical difference between the slope of the standard curve of the target gene primer and that of the GAPDH primer (P>0.05). Amplification efficiency of the five mRNA primers were between 0.85~0.94.

Supplementary Table S3. Statistical analysis for amplification efficiency of miRNA primers

Primer	Slope	95%CI	Constant	Amplification efficiency
cel-miR-238	-1.098	-1.270~-0.927	25.136	0.88
miR-92a-3p	-1.125	-1.354~-0.897	24.642	0.85
miR-486-5p	-1.153	-1.361~-0.945	24.747	0.82

There is no statistical difference between the slope of the standard curve of the target miRNA primer and that of the *cel-miR-238* primer (P>0.05). Amplification efficiency of the three miRNA primers were between 0.82~0.88.

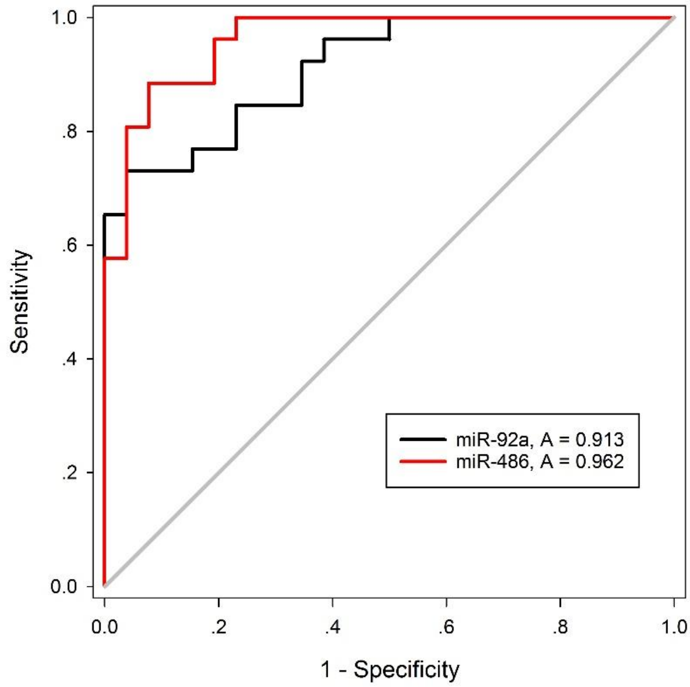

Figure S1. ROC curves of female workers with mercury poisoning

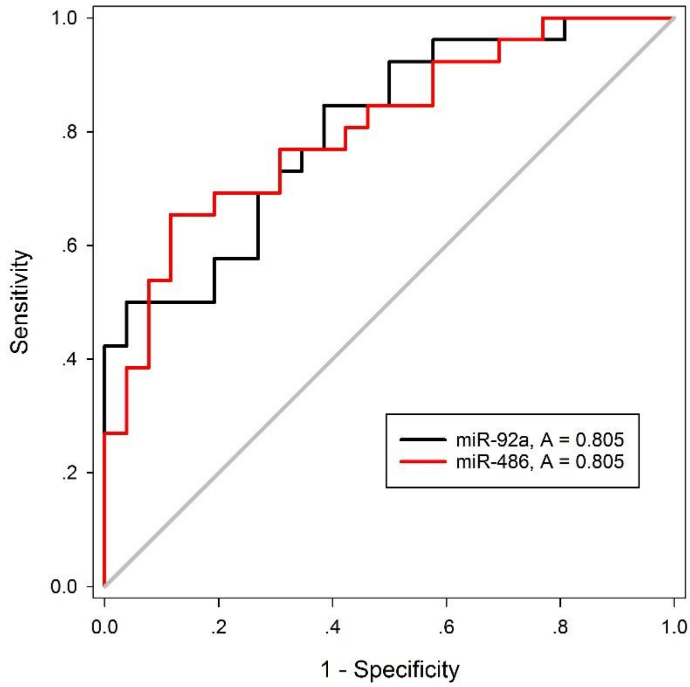

Figure S2. ROC curves of female workers with mercury absorbing
